# Development of Acellular Hepatic Scaffolds Through a Low-Cost Gravity-Assisted Perfusion Decellularization Method

**DOI:** 10.3390/biomimetics10110777

**Published:** 2025-11-15

**Authors:** María Fernanda Duarte-Ortega, Luis Bernardo Enríquez-Sánchez, Manuel David Pérez-Ruiz, Alfredo Nevárez-Rascón, María Alejandra Favila-Pérez, Alva Rocío Castillo-González, Celia María Quiñonez-Flores, Luis Carlos Hinojos-Gallardo, Víctor Adolfo Ríos-Barrera, Carlos Arzate-Quintana

**Affiliations:** 1 Facultad de Medicina y Ciencias Biomédicas, Universidad Autónoma de Chihuahua, Chihuahua 31109, Mexico; 334862@uach.mx (M.F.D.-O.); lbenriquez@uach.mx (L.B.E.-S.); mdperez@uach.mx (M.D.P.-R.); afavila@uach.mx (M.A.F.-P.); acastillo@uach.mx (A.R.C.-G.); cquinonezf@uach.mx (C.M.Q.-F.); 2 Facultad de Odontología, Universidad Autónoma de Chihuahua, Chihuahua 31110, Mexico; alnevarez@uach.mx (A.N.-R.); varios@uach.mx (V.A.R.-B.); 3 Dirección de Investigación y Posgrado, Universidad Autónoma de Chihuahua, Chihuahua 31203, Mexico; lchinojos@uach.mx

**Keywords:** perfusion decellularization, rat liver, acellular scaffolds, tissue engineering, biomimetics

## Abstract

Background: Developing reliable and cost-effective decellularization methods is critical for advancing tissue engineering and regenerative medicine, particularly in regions with limited access to specialized perfusion systems. Methods: This study standardized a gravity-assisted perfusion protocol for rat liver decellularization, designed to operate without pumps or pressurized equipment. Adult Wistar rat livers were processed through a gravity-driven vascular flushing method and compared with a conventional immersion-based protocol. The resulting scaffolds were evaluated by macroscopic inspection, histological staining (Masson’s trichrome), and residual DNA quantification. Results: The gravity-assisted perfusion method achieved more efficient cellular removal and superior preservation of extracellular matrix (ECM) integrity compared with immersion. Residual DNA levels were 3.7 ng/mg in perfused samples, 209.47 ng/mg in immersed samples, and 331.97 ng/mg in controls, confirming a statistically significant reduction (*p* < 0.05). Only the perfused group met the accepted threshold for effective decellularization (<50 ng/mg dry tissue). Histological analysis corroborated these findings, showing the absence of nuclei and the preservation of collagen architecture characteristic of a structurally intact ECM. Conclusions: This low-cost, reproducible, and technically simple system enables the generation of high-quality acellular hepatic scaffolds without mechanical pumps. Its accessibility and scalability make it suitable for laboratories with limited infrastructure and educational settings. Moreover, this gravity-assisted approach provides a foundation for future recellularization and preclinical studies aimed at developing bioengineered liver constructs for regenerative and transplant applications.

## 1. Introduction

Liver transplantation remains the most effective therapeutic option for patients suffering from end-stage liver disease, including cirrhosis, fulminant hepatitis, and hepatocellular carcinoma [1]. Despite major advances in surgical techniques, immunosuppression, and perioperative care, the applicability of liver transplantation is critically constrained by the severe shortage of suitable donor organs [2]. Globally, thousands of patients die each year while on waiting lists, underscoring the magnitude of this clinical challenge [3,4].

For example, in the United States alone, more than 100,000 individuals currently await life-saving organ transplants, and approximately 20 to 30 patients die each day while waiting for a suitable donor [5]. In Mexico, the number of patients on waiting lists for solid-organ transplantation increased from approximately 5000 in 2007 to over 15,000 in 2018, and by early 2022 the figure exceeded 22,000, resulting in a liver transplantation rate below one procedure per million inhabitants—a figure that reflects both the scarcity of multiorgan donors and the insufficient development of comprehensive transplantation programs [6,7]. These figures make the urgency of developing alternative hepatic tissue strategies abundantly clear.

This mismatch between supply and demand leads to long waiting times, lower survival rates, and a significant decline in the quality of life of affected patients. The persistent gap highlights the urgent need for alternative strategies capable of alleviating organ shortages and providing functional hepatic tissue substitutes [1,8,9].

To address this unmet need, researchers have explored a wide range of strategies to generate functional hepatic tissue substitutes or to extend the donor pool. Several approaches have been explored to overcome the limitations of donor availability, ranging from autografts and allografts to isografts and xenografts. Xenotransplantation, in particular, has attracted attention due to the anatomical and physiological similarities between porcine and human livers [10,11]. Nevertheless, xenografts face considerable barriers, including the risk of zoonotic infections, immunological incompatibility, and ethical concerns [2]. Moreover, long-term graft survival has not yet been convincingly demonstrated in clinical contexts, thereby restricting their adoption as a definitive therapeutic solution [12]. Given these persistent challenges, research focus has progressively shifted toward organ bioengineering strategies, which aim to generate functional hepatic constructs tailored to patient-specific needs [3,8].

Among the various approaches explored within this field, one of the most promising is the decellularization and recellularization of biological scaffolds. The principle underlying this approach is biomimetic in nature: the extracellular matrix (ECM) of a native liver provides not only the three-dimensional architecture of the organ but also a complex microenvironment that supports cell attachment, migration, and differentiation [13,14,15]. By carefully removing cellular components while preserving ECM proteins such as laminin, collagen type I/IV, and fibronectin, researchers can obtain scaffolds that retain essential structural and biochemical cues. These scaffolds can subsequently be repopulated with autologous or stem-cell-derived hepatocytes, endothelial cells, and cholangiocytes, with the aim of reconstructing functional hepatic tissue. Importantly, the biomimetic features of decellularized scaffolds—vascular networks, sinusoidal architecture, and mechanical properties—are critical for facilitating perfusion, nutrient delivery, and cell survival after recellularization [16,17,18].

Over the past two decades, several experimental models have demonstrated the feasibility of liver decellularization using both perfusion-based and immersion-based methods in small animals such as rats and rabbits, as well as in larger species including pigs. Perfusion methods generally employ detergents and enzymes delivered through the vascular system to remove cellular debris, while immersion techniques rely on diffusion of these agents into the tissue. Although immersion is technically simpler, it often results in incomplete cell removal and disruption of ECM architecture. In contrast, perfusion decellularization tends to yield more uniform results and superior preservation of structural proteins [19].

From a quality assessment perspective, effective decellularization is defined not only by the removal of cellular material but also by the preservation of extracellular matrix (ECM) integrity. Histological techniques such as hematoxylin and eosin (H&E) staining, Masson’s trichrome, and immunohistochemistry for ECM proteins are commonly employed to confirm decellularization quality, while biochemical assays and mechanical testing provide additional insights into scaffold stability [8]. Quantitative benchmarks typically include residual DNA content below 50 ng per mg of dry tissue, DNA fragment length under 200 base pairs, and minimal disruption of collagen and glycosaminoglycans. Meeting these parameters is essential to minimize immunogenicity and ensure that the scaffold can support subsequent recellularization [20]. However, achieving such high-quality decellularization often requires sophisticated perfusion systems, which introduces new challenges related to cost, accessibility, and technical complexity.

Despite these advances, significant limitations persist. Many perfusion-based decellularization systems rely on peristaltic or pressure-controlled pumps that are expensive and require specialized training to operate. This dependency poses a substantial barrier for laboratories and healthcare systems functioning under constrained budgets, particularly in developing regions such as Latin America. Consequently, there is a growing interest in designing low-cost, easy-to-reproduce perfusion systems capable of achieving comparable decellularization outcomes while minimizing technical and financial demands [21]. In this context, gravity-assisted perfusion offers a simple yet effective alternative. By exploiting hydrostatic pressure as the driving force for vascular flushing, it enables controlled flow without the need for mechanical pumps or pressurized circuits. This approach is economical, reproducible, and scalable, thereby expanding accessibility to organ bioengineering research and training in resource-limited environments. Furthermore, the mechanical simplicity of this setup lends itself to custom fabrication through computer-aided design and 3D printing [22], which could facilitate local production of perfusion prototypes adapted to different organ sizes and experimental needs [19,23].

Previous reports have suggested that gravity-based perfusion systems can generate scaffolds of acceptable quality; however, comparative analyses with immersion methods and standardized evaluation of ECM preservation remain limited [24]. Establishing reliable, low-cost, and reproducible protocols could therefore represent a transformative step toward democratizing organ bioengineering. Such innovations not only enhance experimental feasibility but also hold promise for translational applications, including disease modeling, drug testing, and potentially, preclinical transplantation.

In this context, the present exploratory study aimed to standardize a gravity-assisted perfusion decellularization method for rat liver as a low-cost and reproducible alternative to conventional perfusion systems. The proposed approach was compared with a traditional immersion protocol and evaluated through morphological, histological, and DNA analyses, with emphasis on extracellular matrix preservation. By demonstrating that effective decellularization can be achieved using only hydrostatic pressure, this work introduces a simple and scalable strategy that expands access to hepatic scaffold production, particularly in laboratories with limited resources and infrastructure.

## 2. Materials and Methods

### 2.1. Animal Model and Ethical Considerations

Four adult male Wistar rats (600–650 g) were used in this study. The animals were housed in the institutional animal facility under controlled environmental conditions (22 ± 2 °C, 50 ± 10% relative humidity) with a 12 h light–dark cycle and free access to standard laboratory chow and sterile water ad libitum. One liver was used as an untreated control, one was processed through the immersion protocol, and two through the gravity-assisted perfusion protocol to ensure reproducibility within that condition. For DNA quantification, two independent samples were obtained from the perfusion group (one from each liver), while two samples from distinct regions were taken from the same control and immersion livers to account for intra-organ variability.

Euthanasia was performed by inhalation of sevoflurane, following the Mexican Official Standard NOM-062-ZOO-1999 [25] for humane treatment of laboratory animals. No experimental interventions were performed; organs were collected post-mortem for decellularization assays only. Biological remains were disposed of as regulated biomedical waste according to NOM-087-SEMARNAT-SSA1-2002 [26], using certified RPBI containers (A1, S.A. de C.V., Ciudad de México, Mexico).

The protocol was reviewed and approved by the Institutional Committee for the Care and Use of Laboratory Animals (CICUAL), Faculty of Medicine and Biomedical Sciences, Universidad Autónoma de Chihuahua. Approval was granted under registration number CI-060-23.

### 2.2. Experimental Design

The study was designed as an experimental, longitudinal, analytical, comparative, and non-blinded investigation. Two decellularization protocols (gravity-assisted perfusion and immersion) were applied to rat liver samples, and outcomes were compared based on DNA quantification and histological evaluation.

### 2.3. Liver Harvesting Procedure

Wistar rats were anesthetized by inhalation of sevoflurane and subsequently euthanized. Death was confirmed before proceeding with the surgical procedure. A thoracoabdominal incision was performed to expose the cavity. Ten units of heparin were injected directly into the heart through the diaphragm [27] and a two-minute interval was allowed [28].

A midline supra- and infraumbilical incision was made, followed by stepwise dissection until the abdominal cavity was exposed. The liver was identified, and the hepatic hilum was dissected to visualize the bile duct and portal vein. The portal vein was cannulated, the inferior vena cava was ligated, and the superior vena cava was separated from the diaphragm and heart before being cannulated and sectioned proximally. The organ was gently released from its attachments and excised. Subsequently, phosphate-buffered saline (PBS) was injected through the fixed suprahepatic inferior vena cava [29]. Finally, the liver was placed in a Petri dish containing PBS and stored at −80 °C until further use [27].

### 2.4. Decellularization Protocols

#### 2.4.1. Immersion Method

During the immersion decellularization process, whole rat livers were completely submerged in the corresponding chemical solutions following a sequential washing and treatment scheme. Initially, the organs were placed in phosphate-buffered saline (PBS) for 24 h to remove blood residues and soluble materials. Subsequently, they were exposed to increasing concentrations of sodium dodecyl sulfate (SDS)—0.01%, 0.1%, 0.2%, and 0.5%—for specific periods ranging from 1 h and 20 min to 4 h, in order to gradually remove cellular components while minimizing structural damage to the ECM. After detergent exposure, the samples were rinsed with distilled water for 1 h to eliminate residual SDS, followed by treatment with 1% Triton X-100 for 4 h to solubilize lipid membranes and remaining cytoplasmic debris. Finally, the tissues were washed again with PBS for 1 h, completing a total processing time of approximately 47 h [2].

#### 2.4.2. Gravity-Assisted Perfusion Method

**Figure 1:**
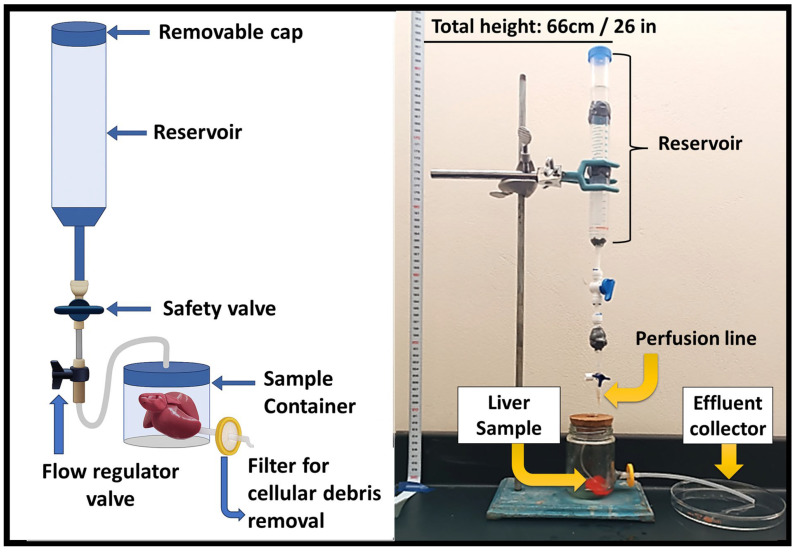
Schematic (**left**) and experimental setup (**right**) of the gravity-assisted perfusion system used for rat liver decellularization. The setup operates by gravitational flow from a 66 cm reservoir through a controlled perfusion line into the hepatic vasculature, allowing for passive detergent circulation and effluent collection.

The perfusion decellularization protocol involved the continuous passage of the same chemical solutions through the portal vein, hepatic artery, and hepatic vein, ensuring homogeneous reagent distribution throughout the hepatic parenchyma [28]. The setup consisted of an elevated reservoir containing the detergent solution, connected via silicone tubing to a cannula inserted into the portal vein of the liver. The effluent exited through an outlet tube attached to the hepatic vein and drained into a waste collection flask. This gravity-assisted configuration enabled passive perfusion driven solely by hydrostatic pressure, eliminating the need for pump-generated flow. The procedure began with PBS perfusion for 10 h, followed by SDS solutions at concentrations of 0.01%, 0.1%, 0.2%, and 0.5% for 15, 2, 3, and 3 h, respectively. Subsequently, a 1% Triton X-100 solution was perfused for 16 h to remove lipid residues, followed by final rinsing with PBS (3 h) and distilled water (1 h). The total processing time was approximately 53 h. A detailed summary of immersion and perfusion steps, including reagent concentrations and exposure times, is provided in Supplementary Table S1, and a schematic representation of the gravity-driven perfusion system is shown in Figure 1.

#### 2.4.3. DNA Extraction and Quantification

**Figure 2:**
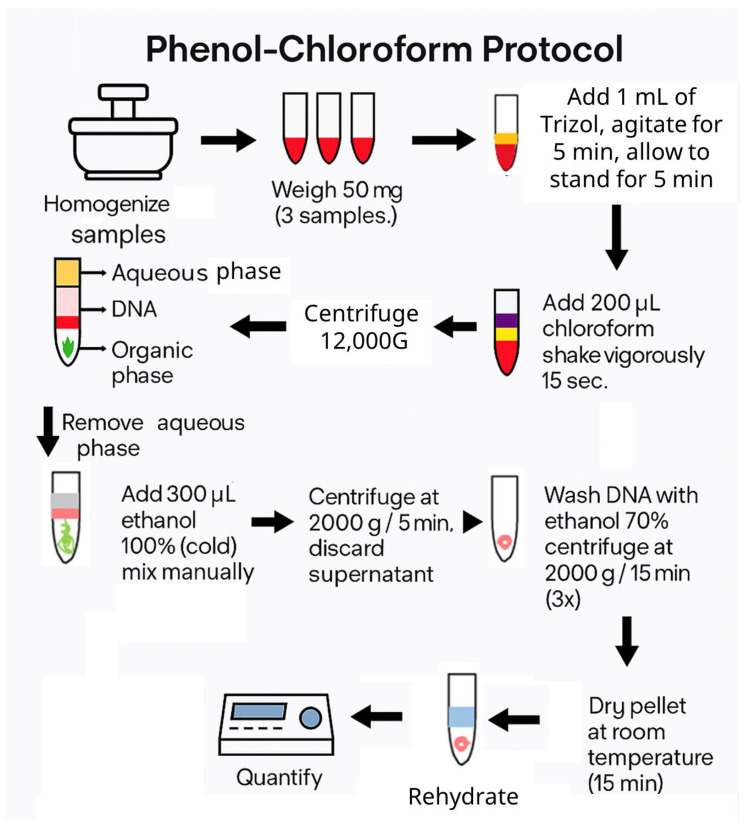
Flowchart of the phenol–chloroform DNA quantification protocol. The diagram summarizes the main steps involved in the extraction and purification of genomic DNA from liver tissue, including sample maceration, Trizol treatment, chloroform extraction, ethanol precipitation, washing, pellet drying, and spectrophotometric quantification.

Genomic DNA was extracted using a modified phenol–chloroform protocol adapted to the present methodology, employing Trizol reagent (Invitrogen, Waltham, MA, USA) as the main component. Trizol is a phenol-based solution commonly used for RNA isolation, containing phenol and hydroxyquinoline as RNase inhibitors, among other active compounds. Approximately 50 mg of decellularized liver tissue was processed for each sample. The extraction procedure consisted of tissue homogenization in 1 mL of Trizol, followed by phase separation with 200 μL of chloroform. The resulting aqueous phase was subsequently purified through sequential washes with 300 μL of 100% ethanol and 1 mL of 70% ethanol to remove residual contaminants. DNA was finally eluted in 50 μL of distilled or injectable water and quantified by measuring absorbance at 260/280 nm using an Eppendorf BioSpectrometer Basic (Eppendorf SE, Hamburg, Germany) (Figure 2).

#### 2.4.4. Histological Evaluation

**Figure 3:**
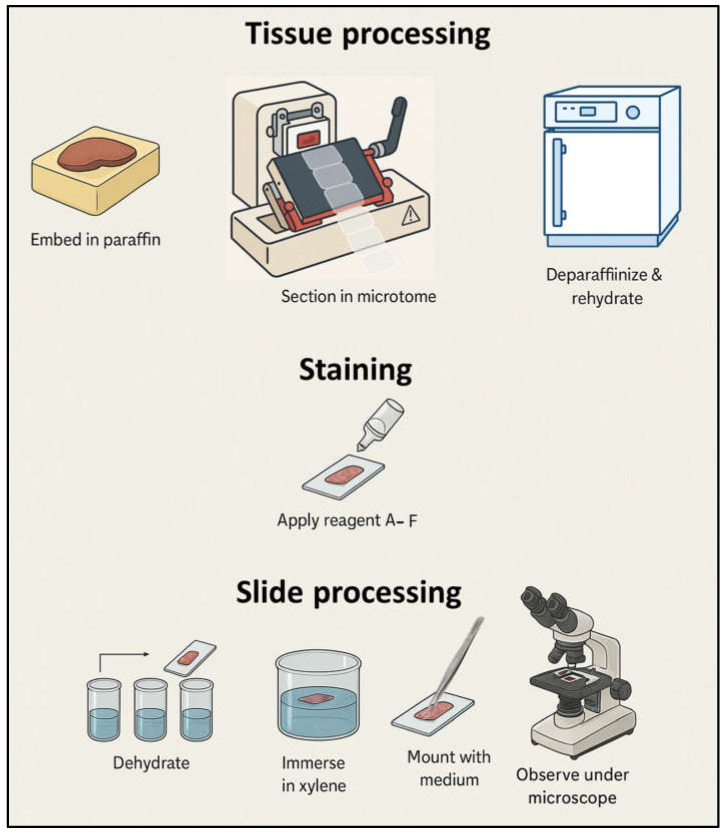
Schematic representation of the histological processing and Masson’s trichrome staining procedure. The workflow illustrates the sequential steps of tissue fixation, dehydration, paraffin embedding, microtome sectioning, deparaffinization, rehydration, and staining with reagents A–F. Following dehydration and clearing, the sections were mounted with permanent medium and examined under an optical microscope at 100× and 400× magnifications to assess tissue morphology and extracellular matrix preservation.

Histological analysis was performed using Masson’s trichrome staining with aniline blue (PanReac AppliChem, Darmstadt, Germany), which allows differentiation of nuclei, cytoplasm, connective tissue, keratin, and collagen fibers. Tissue sections were paraffin-embedded, cut at 5 µm, deparaffinized, and rehydrated using standard histological procedures. The protocol was optimized to enhance collagen visualization in decellularized matrices by adjusting staining times for the aniline blue and acid fuchsin reagents, ensuring consistent contrast between cellular remnants and extracellular fibers. After staining, sections were dehydrated through graded ethanol, cleared in xylene, and mounted with a permanent medium. Images were obtained under a standard optical microscope equipped with a digital camera at 100× and 400× magnifications (Figure 3).

## 3. Results

### 3.1. Macroscopic Analysis

**Figure 4:**
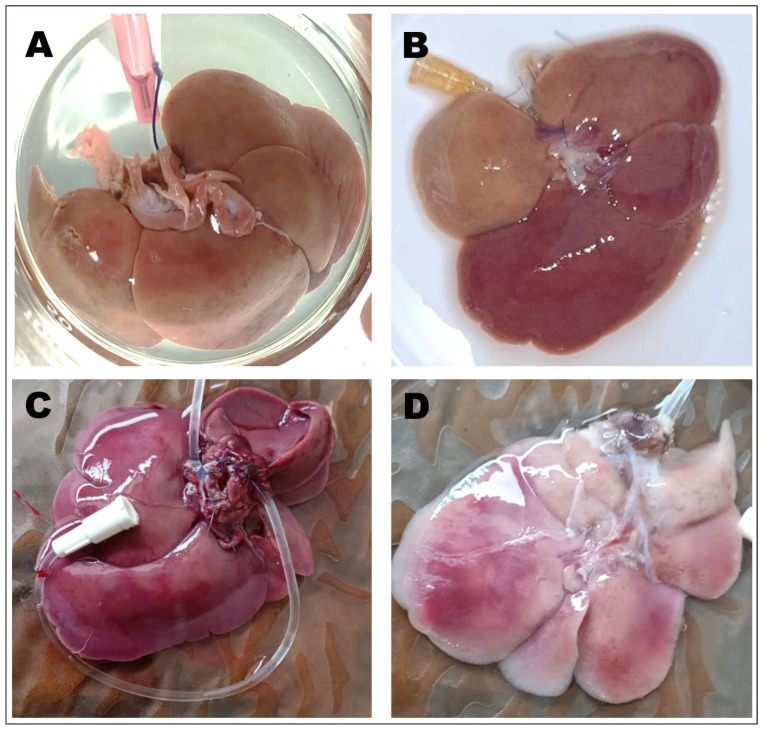
Macroscopic view of rat liver prior (**A**) and after (**B**) immersion decellularization, showing partial discoloration and heterogeneous opacity. And a second sample before (**C**) and after (**D**) gravity-assisted perfusion decellularization. The perfused sample exhibits a lighter, translucent appearance, indicating effective removal of cellular material.

Macroscopic inspection revealed evident morphological differences between untreated and processed rat liver samples. The native liver (Figure 4A,C) exhibited a homogeneous dark reddish coloration and a compact, well-vascularized parenchymal structure characteristic of fresh hepatic tissue. In contrast, samples subjected to immersion and gravity-assisted perfusion (Figure 4B,D) showed progressive loss of pigmentation and increased translucency, reflecting the depletion of hemoglobin and cellular material during detergent exposure. The perfused liver in particular exhibited a pale pink to whitish tone with visible vascular channels and reduced opacity, indicating more extensive cell removal while maintaining the overall lobular architecture. These macroscopic transformations are consistent with effective decellularization, as the loss of pigmentation and increased translucency are commonly associated with the removal of cellular components while maintaining the gross architecture of the extracellular matrix.

### 3.2. DNA Quantification

**Figure 5:**
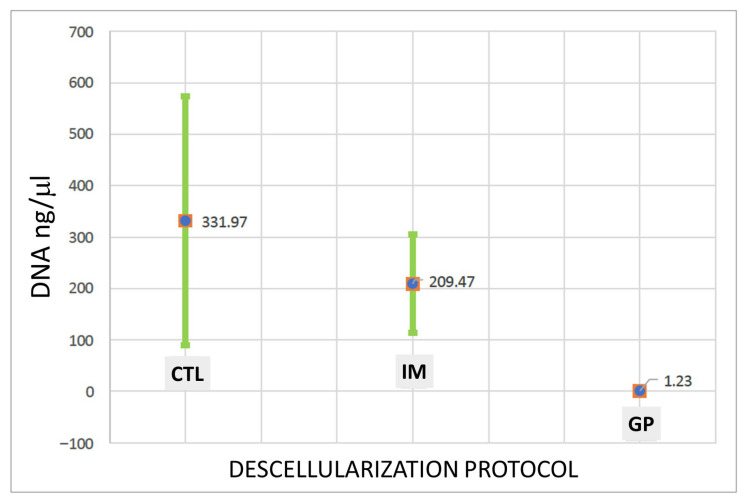
DNA concentration (ng/μL) measured in control (CTL), immersion (IM), and gravity-assisted perfusion (GP) groups. Three replicates were analyzed per group, and concentrations were quantified by spectrophotometry. The graph displays mean values ± SD for each protocol.

DNA quantification showed a progressive reduction across the experimental groups (Figure 5). The control samples contained 331.97 ng/mg of DNA, while the immersion-treated samples decreased to 209.47 ng/mg, remaining above the accepted limit of 50 ng/mg for effective decellularization. The gravity-assisted perfusion group exhibited the lowest value, 1.23 ng/mg, confirming a significant reduction compared to the other protocols (*p* < 0.05).

### 3.3. Histological Analysis

Histological evaluation was performed using Masson’s trichrome staining to assess the extent of cellular removal and ECM preservation.

**Figure 6:**
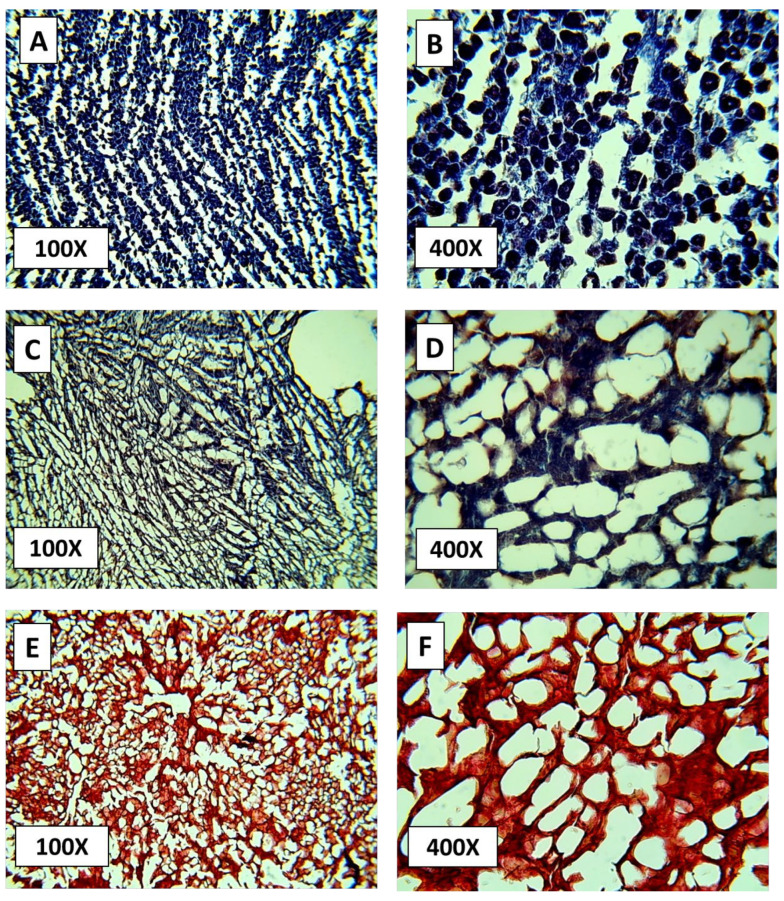
Representative histological images of rat liver sections stained with Masson’s trichrome at 100× and 400× magnifications. (**A**,**B**) Native control tissue showing intact hepatic architecture. (**C**,**D**) Immersion-treated tissue displaying partial cell removal. (**E**,**F**) Gravity-assisted perfusion-treated tissue showing effective decellularization and preserved extracellular matrix structure.

In the control tissue, hepatic cords containing abundant hepatocytes were evident. At 100× magnification, nuclei and cytoplasm appeared dark blue, and connective tissue regions were visible around blood vessels, particularly near the central vein of hepatic lobules (Figure 6A). At higher magnification (400×), hepatocytes exhibited dark purple cytoplasm with lighter contrasting nuclei, while perivascular connective tissue displayed a distinct bluish hue (Figure 6B).

In the immersion method, residual cellularity persisted within the stromal network. At 100×, cells showed heterogeneous morphology with violet to burgundy cytoplasmic staining and purple nuclei (Figure 6C). Connective tissue elements were mainly confined to vascular walls. At 400×, small clusters of degenerated hepatocytes remained, and the overall stromal continuity appeared irregular, indicating incomplete decellularization (Figure 6D).

In the perfusion method, hepatocytes were largely absent, leaving a coarse stromal mesh. At 100×, the tissue displayed a uniform reddish-brown appearance with minimal nuclear remnants (Figure 6E). At 400×, the translucent matrix and preserved vascular outlines indicated efficient cell removal while maintaining the general framework of the ECM (Figure 6F). Although Masson’s trichrome is not specific for collagen identification, the observed structural integrity suggests that the underlying scaffold remained morphologically intact.

## 4. Discussion

This exploratory study successfully standardized a gravity-assisted perfusion method for rat liver decellularization, demonstrating that effective cell removal and preservation of ECM components can be achieved without the use of mechanical pumps or complex bioreactors. The approach validated our initial hypothesis, confirming that hydrostatic pressure generated by gravity is sufficient to maintain a controlled and uniform flow capable of achieving consistent decellularization throughout hepatic tissue. However, as an exploratory proof of concept, the study was limited by a small sample size (*n* = 4), which restricts statistical generalization. Despite this limitation, the consistency of the results across replicates supports the technical feasibility and reproducibility of the gravity-assisted perfusion approach, providing a solid foundation for future large-scale validation and recellularization studies.

Quantitative DNA analysis provided objective evidence of the protocol’s efficiency. Control samples exhibited an average residual DNA concentration of 331.97 ng/mg of dry tissue, consistent with normal cellular density. Immersion-treated samples showed partial reduction to 209.47 ng/mg, remaining above the internationally accepted threshold of 50 ng/mg for effective decellularization [20]. In contrast, the gravity-assisted perfusion scaffolds presented markedly lower residual DNA levels (1.23 ng/mg), well within the established limit for minimal immunogenicity.

Masson’s trichrome staining provided clear evidence of the decellularization efficacy across the experimental groups. In the control tissue, the intense blue coloration of nuclei and cytoplasm confirmed the presence of abundant hepatocytes with preserved architecture, while dense collagen fibers appeared distinctly stained in royal blue around the vascular structures. In contrast, liver sections processed by immersion showed partial cellular removal, with residual hepatocytes of heterogeneous morphology and limited collagen distribution, particularly around vascular walls. These findings indicate an incomplete decellularization process, likely due to the restricted diffusion of detergents into the parenchyma [30].

Conversely, samples subjected to gravity-assisted perfusion exhibited minimal nuclear staining and a markedly translucent stromal mesh, indicating substantial removal of cellular components. The residual matrix retained its fibrous framework with preserved vascular outlines, consistent with efficient decellularization [8,16]. Although Masson’s trichrome staining effectively revealed differences in tissue morphology and overall ECM preservation [19], it does not specifically identify collagen or nuclear material. Therefore, future stages of this ongoing project will incorporate complementary H&E and additional histochemical analyses to confirm the absence of nuclei and to better characterize ECM components, including collagen distribution and integrity.

The macroscopic transformations observed in perfused livers were also consistent with these histological results. After treatment, organs became pale and translucent, with visible vascular channels replacing the dense reddish parenchyma of native liver tissue. Such changes reflect the progressive removal of hemoglobin and cellular material while maintaining the organ’s gross structure—an indicator of effective decellularization.

Beyond confirming the technical feasibility of this gravity-driven system, the present work highlights its practical relevance for laboratories and healthcare institutions operating under limited-resource conditions. The system’s simplicity—requiring no pumps, sensors, or pressurized circuits—makes it suitable for low-cost implementation and for educational purposes in tissue engineering courses [31,32]. Moreover, its modular design can be adapted using 3D-printed components, allowing reproducibility and scalability for different organ sizes or research needs [22]. This approach aligns with emerging trends in advanced manufacturing and 4D printing technologies for tissue engineering, as demonstrated by Choudhury et al. [33].

The preservation of ECM integrity achieved through this simplified setup is crucial for future recellularization and regenerative applications [17,32]. More importantly, this model supports a paradigm shift toward accessible and sustainable biofabrication. It demonstrates that high-quality tissue engineering outcomes do not necessarily depend on high-cost infrastructure, but rather on well-conceived experimental design and reproducible methodology [34,35].

Overall, the gravity-assisted perfusion technique represents a robust, accessible, and sustainable approach to organ decellularization. It aligns with the principles of biomimetic engineering by integrating biological functionality with technological simplicity. Future studies should focus on optimizing flow dynamics, scaling the model to larger organs, and evaluating recellularization potential, thereby advancing the development of bioengineered liver constructs for translational and preclinical applications.

Future studies will focus on expanding the number of biological replicates to strengthen statistical validity and reproducibility. Complementary histological analyses, including hematoxylin–eosin staining, will be incorporated to confirm the absence of nuclear material and provide a more detailed assessment of ECM composition. Additionally, recellularization assays using hepatocyte cultures are planned to evaluate the functional potential of the acellular scaffolds. These next steps aim to consolidate the gravity-assisted perfusion approach as a reliable, low-cost platform for hepatic tissue bioengineering and preclinical regenerative applications.

## 5. Conclusions

This study successfully standardized a gravity-assisted perfusion method for rat liver decellularization, demonstrating that effective cell removal and preservation of the ECM can be achieved without the use of mechanical pumps or complex bioreactors. Quantitative analysis confirmed a marked reduction in residual DNA—from 331.97 ng/mg in controls and 209.47 ng/mg in immersion-treated samples to 1.23 ng/mg in perfused scaffolds—meeting the international threshold for effective decellularization (<50 ng/mg dry tissue). Histological findings corroborated these results, showing uniform cellular removal and preserved vascular architecture.

Perfusion proved significantly more effective than immersion, which exhibited heterogeneous decellularization due to limited detergent diffusion in thick parenchymal tissue. Although the perfusion process required extended working times, avoiding freeze–thaw cycles improved scaffold quality and reduced ECM disruption.

A key limitation of this exploratory study was the small sample size (n = 4), which restricts statistical generalization; however, results were consistent across replicates. Future work will incorporate hematoxylin–eosin staining and expand sample numbers to validate reproducibility and assess recellularization potential.

Overall, this low-cost and technically simple perfusion system represents an accessible strategy for producing acellular hepatic scaffolds in laboratories with limited resources, providing a foundation for future preclinical studies and scalable bioengineering applications.

## Data Availability

The data presented in this study are available in the article.

## Supplementary Materials

The following supporting information can be downloaded at https://www.mdpi.com/article/10.3390/biomimetics10110777/s1, Table S1: Decellularization process for each protocol.

## References

[1] Nair D.G., Weiskirchen R. (2023). Recent Advances in Liver Tissue Engineering as an Alternative and Complementary Approach for Liver Transplantation. Curr. Issues Mol. Biol..

[2] Dai Q., Jiang W., Huang F., Song F., Zhang J., Zhao H. (2022). Recent Advances in Liver Engineering with Decellularized Scaffold. Front. Bioeng. Biotechnol..

[3] Meng F., Assiri A., Dhar D., Broering D. (2017). A Promising Approach to Develop Functional Liver Surrogates. Liver Int..

[4] De Kock J., Ceelen L., De Spiegelaere W., Casteleyn C., Claes P., Vanhaecke T., Rogiers V. (2011). Simple and Quick Method for Whole-Liver Decellularization: A Novel In Vitro Three-Dimensional Bioengineering Tool?. Arch. Toxicol..

[5] United Network for Organ Sharing (UNOS) Organ Donation Statistics. U.S. Department of Health and Human Services. https://www.organdonor.gov/learn/organ-donation-statistics.

[6] González F.X., Cordero E., López R. (2016). Deceased Donation and Organ Transplantation in Mexico. Transplant. Proc..

[7] Centro Nacional de Trasplantes (CENATRA) (2024). Informe Estadístico de Donación y Trasplantes en México. https://www.gob.mx/cenatra.

[8] Morawski M., Krasnodębski M., Rochoń J., Kubiszewski H., Marzęcki M., Topyła D., Murat K., Staszewski M., Szczytko J., Maleszewski M. (2025). Decellularized Liver Matrices for Expanding the Donor Pool—An Evaluation of Existing Protocols and Future Trends. Biomolecules.

[9] He M., Callanan A. (2012). Comparison of Methods for Whole-Organ Decellularization in Tissue Engineering of Bioartificial Organs. J. Tissue Eng. Part B Rev..

[10] Cooper D.K.C., Ekser B., Tector A.J. (2015). A Brief History of Clinical Xenotransplantation. Int. J. Surg..

[11] Lupon E., Acun A., Taveau C.B., Oganesyan R., Lancia H.H., Andrews A.R., Randolph M.A., Cetrulo C.L., Lellouch A.G., Uygun B.E. (2024). Optimized Decellularization of a Porcine Fasciocutaneous Flap. Bioengineering.

[12] Van Hengel E.V.A., van der Laan L.J.W., de Jonge J., Verstegen M.M.A. (2025). Towards Safety and Regulation Criteria for Clinical Applications of Decellularized Organ-Derived Matrices. Bioengineering.

[13] Struecker B., Butter A., Hillebrandt K., Polenz D., Reutzel-Selke A., Tang P., Lippert S., Leder A., Rohn S., Geisel D. (2017). Improved Rat Liver Decellularization by Arterial Perfusion under Oscillating Pressure Conditions. J. Tissue Eng. Regen. Med..

[14] Gomes K.T., Prasad P.R., Sandhu J.S., Kumar A., Kumar N.A.N., Shridhar N.B., Bisht B., Paul M.K. (2025). Decellularization Techniques: Unveiling the Blueprint for Tracheal Tissue Engineering. Front. Bioeng. Biotechnol..

[15] Xu M.S., D’Elia A., Hadzimustafic N., Adil A., Karoubi G., Waddell T.K., Haykal S. (2024). Bioengineering of Vascularized Porcine Flaps Using Perfusion–Recellularization. Sci. Rep..

[16] Croce S., Peloso A., Zoro T., Avanzini M.A., Cobianchi L. (2019). A Hepatic Scaffold from Decellularized Liver Tissue. Biomolecules.

[17] Toprakhisar B., Verfaillie C.M., Kumar M. (2023). Advances in Recellularization of Decellularized Liver Grafts with Different Liver (Stem) Cells: Towards Clinical Applications. Cells.

[18] Meșină M., Mîndrilă I., Meșină C., Obleagă C.V., Istrătoaie O. (2019). A Perfusion Decellularization Heart Model—An Interesting Tool for Cell–Matrix Interaction Studies. J. Mind Med. Sci..

[19] Shevchuk O.I., Korcheva V.V., Moskalenko N.S., Kyryk V.M., Kot K.V., Krasnienkov D.S. (2025). Application of Decellularization Methods for Scaffold Production: Advantages, Disadvantages, Biosafety, and Modifications. Front. Bioeng. Biotechnol..

[20] Diedrich A.M., Daneshgar A., Tang P., Klein O., Mohr A., Onwuegbuchulam O.A., von rueden S., Menck K., Bleckmann A., Juratli M.A. (2024). Proteomic Analysis of Decellularized Mice Liver and Kidney Extracellular Matrces. J. Biol. Eng..

[21] Taylor D.A., Kren S.M., Rhett K., Robertson M.J., Morrissey J., Rodriguez O.E., Virk H., Chacon-Alberty L., da Costa E.C., Mesquita F.C.P. (2021). Characterization of Perfusion-Decellularized Whole Animal Body, Isolated Organs, and Multi-Organ Systems for Tissue Engineering Applications. Physiol. Rep..

[22] Shi W., Zhang Z., Wang X. (2024). The Prospect of Hepatic Decellularized Extracellular Matrix as a Bioink for Liver 3D Bioprinting. Biomolecules.

[23] Morales-Guerrero N.A., Varela-Echavarría A., Lozano-Flores C., Vázquez-Cuevas F.G., Velázquez-Miranda E., Reyes-López J.V., García-Solís P., Solís-S J.C., Hernandez-Montiel H.L. (2022). A New Strategy for the Decellularization of Whole Organs by Hydrostatic Pressure. Biotechnol. Prog..

[24] Zubarevich A., Osswald A., Amanov L., Rad A.A., Schmack B., Ruhparwar A., Weymann A. (2023). Development and Evaluation of a Novel Combined Perfusion Decellularization Heart–Lung Model for Tissue Engineering of Bioartificial Heart–Lung Scaffolds. Artif. Organs.

[25] (1999). Especificaciones Técnicas Para la Producción, Cuidado y uso de los Animales de Laboratorio.

[26] (2002). Protección Ambiental—Residuos Peligrosos Biológico-Infecciosos—Clasificación y Especificaciones de Manejo.

[27] Geerts S., Ozer S., Jaramillo M., Yarmush M.L., Uygun B.E. (2016). Nondestructive Methods for Monitoring Cell Removal During Rat Liver Decellularization. Tissue Eng. Part C Methods.

[28] Uygun B.E., Price G., Saeidi N., Izamis M.-L., Berendsen T., Yarmush M., Uygun K. (2011). Decellularization and Recellularization of Whole Livers. J. Vis. Exp..

[29] Nari G.A., Cid M., Comín R., Reyna L., Juri G., Taborda R., Salvatierra N.A. (2013). Preparación de una matriz extracelular tridimensional por descelularización de hígados de conejos. Rev. Esp. Enferm. Dig..

[30] Allu I., Sahi A.K., Koppadi M., Gundu S., Sionkowska A. (2023). Decellularization Techniques for Tissue Engineering: Towards Replicating Native Extracellular Matrix Architecture in Liver Regeneration. J. Funct. Biomater..

[31] Nicholls D.L., Rostami S., Karoubi G., Haykal S. (2022). Perfusion Decellularization for Vascularized Composite Allotransplantation. SAGE Open Med..

[32] Neishabouri A., Soltani Khaboushan A., Daghigh F., Kajbafzadeh A.-M., Majidi Zolbin M. (2022). Decellularization in Tissue Engineering and Regenerative Medicine: Evaluation, Modification, and Application Methods. Front. Bioeng. Biotechnol..

[33] Choudhury D., Joshi A., Baghel V.S., Ananthasuresh G.K., Asthana S., Homer-Vanniasinkam S., Chatterjee K. (2024). Design-encoded dual shape-morphing and shape-memory in 4D printed polymer parts toward cellularized vascular grafts. J. Mater. Chemestry B.

[34] Almeida G.H.D.R., da Silva-Júnior L.N., Gibin M.S., dos Santos H., Horvath-Pereira B.d.O., Pinho L.B.M., Baesso M.L., Sato F., Hernandes L., Long C.R. (2023). Perfusion and Ultrasonication Produce a Decellularized Porcine Whole-Ovary Scaffold with a Preserved Microarchitecture. Cells.

[35] Mir T.A., Alzhrani A., Nakamura M., Iwanaga S., Wani S.I., Altuhami A., Kazmi S., Arai K., Shamma T., Obeid D.A. (2023). Whole Liver Derived Acellular Extracellular Matrix for Bioengineering of Liver Constructs: An Updated Review. Bioengineering.

