# Supplementary material for: Development of Acellular Hepatic Scaffolds Through a Low-Cost Gravity-Assisted Perfusion Decellularization Method
Source: Biomimetics (Basel). 2025 Nov 15;10(11):777. doi: 10.3390/biomimetics10110777 (PMC12650586; doi:10.3390/biomimetics10110777)
Supplement: Supplementary file 1 [file biomimetics-10-00777-s001.zip › biomimetics-3960086-supplementary.pdf]

Supplementary Materials

Table S1. Decellularization process for each protocol.

**Protocol 1 (Immersion)**		**Protocol 2 (Perfusion)**	
**Reagent**	Time (h)	Reagent	Time (h)
**PBS**	24	PBS	10
**SDS 0.01%**	4	SDS 0.01%	15
**SDS 0.1%**	4	SDS 0.1%	2
**SDS 0.2%**	4	SDS 0.2%	3
**SDS 0.5%**	1 h 20 min	SDS 0.5%	3
**Distilled water (wash)**	1	Triton X-100 1%	16
**Triton X-100 1%**	8	PBS 1%	3
**PBS 1%**	1	Distilled water	1
**Total time (h)**	47.2	Total time (h)	53

Note: The exposure times for each reagent differed between the immersion and perfusion protocols because the visual progress of decellularization was not comparable between methods. The immersion process exhibited slower and less uniform tissue clearing, requiring longer exposure to achieve partial cell removal, whereas perfusion achieved faster and more homogeneous detergent distribution through vascular conduits. The protocol durations were therefore adjusted empirically based on macroscopic observation of tissue transparency and detergent penetration.
